# Engagement in video and audio narratives: contrasting self-report and physiological measures

**DOI:** 10.1038/s41598-020-68253-2

**Published:** 2020-07-09

**Authors:** Daniel C. Richardson, Nicole K. Griffin, Lara Zaki, Auburn Stephenson, Jiachen Yan, Thomas Curry, Richard Noble, John Hogan, Jeremy I. Skipper, Joseph T. Devlin

**Affiliations:** 1 Experimental Psychology, University College London, London, UK; 2 Audible, London, UK

**Keywords:** Psychology, Human behaviour

## Abstract

Stories play a fundamental role in human culture. They provide a mechanism for sharing cultural identity, imparting knowledge, revealing beliefs, reinforcing social bonds and providing entertainment that is central to all human societies. Here we investigated the extent to which the delivery medium of a story (audio or visual) affected self-reported and physiologically measured engagement with the narrative. Although participants self-reported greater involvement for watching video relative to listening to auditory scenes, stronger physiological responses were recorded for auditory stories. Sensors placed at their wrists showed higher and more variable heart rates, greater electrodermal activity, and even higher body temperatures. We interpret these findings as evidence that the stories were more cognitively and emotionally engaging at a physiological level when presented in an auditory format. This may be because listening to a story, rather than watching a video, is a more active process of co-creation, and that this imaginative process in the listener’s mind is detectable on the skin at their wrist.

## Introduction

Stories help us make sense of the world. Narratives provide links to traditions, legends, archetypes, myths, and symbols and help connect us to others by forming and stabilizing social bonds, by reinforcing and enhancing the group’s memory, and by providing shared entertainment. Our oldest narratives date back many thousands of years and pre-date the advent of writing. For the majority of human history, stories were synonymous with the oral tradition; audiences listened to a story teller imparting a tale. In modern cultures, stories are just as important but now are delivered in a variety of mediums including written books (both physical and digital), videos (TV and films), and as auditory narratives. Here we investigated the extent to which the medium of a story (audio or visual) affected one’s engagement with the narrative.

“Engagement” is construed very differently across the literature^1–4^. In some cases, it refers to cognitive operations such as attention, effort or agency when performing a task^1,2^ while in others it refers more generally to participation in activities^3,4^. In this paper, we operationalize engagement in two ways: self-reported engagement of a narrative experience and physiological engagement as an indirect measure of the mental processing that generated that experience.

A good story takes the listener on a journey, evoking cognitive and emotional responses such that the listener experiences the story through a process of mental simulation of the people, events, actions, places and emotions from the narrative, as if these were being experienced directly^5–7^. Indeed, there is evidence that narratives recreate a similar pattern of brain activity in the listener that was produced by the storyteller. Silbert et al.^8^ used functional magnetic resonance imaging (fMRI) to scan the brain of a volunteer speaking a 15-min personal story. Another set of volunteers then listened to this story while having their brains scanned. The authors identified the set of brain regions engaged by these tasks and found widespread coupling between activity in the speaker’s brain and that in the listeners’ brains. In other words, the act of listening to the narrative recreated the same basic pattern of brain activity as telling the story. This could mean that listeners share the same mental representations as the speaker^9,10^, but at a minimum it demonstrates that listening to the story produces similar neural processing, which may suggest it is similar to experiencing the speaker’s memory of the events. Moreover, activation was not limited to regions of the brain classically related to language, but also involved emotional, sensory and motor systems consistent with the notion that at some level, the listener actually experiences the story.

Historically, story-telling relied primarily on spoken language, and then more recently on written language, but in the modern era video has emerged as a major narrative tool as well. The main difference between these channels is the information they provide. Spoken words come in a single modality, namely audition, and have a very abstract relation to the content of the narrative. Consider a story that contains the sentence: “The was house ablaze.” A listener will correctly interpret this to mean that the house was on fire and possibly imagine what it might be like but the actual physical stimulus—in this case, changes in acoustic energy over time—is unrelated to the content being conveyed except through the interpretation of language. Video, on the other hand, is more closely related to the content. Seeing a video of a burning house, hearing the sounds of the fire—these are physical stimuli that directly convey the information without interpretation and without language. In other words, because of their different information content, these two channels require very different processing despite the fact that they can convey identical narrative content. Oral and written stories require a more active processing in the sense that the listener/reader reconstructs a personalized interpretation of the narrative. In contrast, watching video is a more passive process due to the fact that there is less scope for personal interpretation. Indeed, Jajdelska et al.^11^ recently reviewed a wide field of evidence investigating the differences between narrative processing of spoken or written stories, and moving images. They concluded that, ‘verbal narrative generates more diverse responses than moving image narrative,’ and recommend that future research focuses on differences in neural mechanism between the two. In this paper, we rise to that challenge, and predict that different levels of mental processing of auditory and video narratives will be reflected in different levels of physiological activity, specifically in heart rate, electrodermal activity and body temperature.

In its simplest form, increased heart rate is an indicator of increased effort and serves as an indirect measure of cognitive and emotional engagement. Changes in heart rate have been linked to increased information processing demands and/or greater mental effort^12–14^. Linking heart rate to specific cognitive states, however, is not straightforward. Andreassi^12^ claimed that heart rates increase when people focus more on internal information and less on the external environment while Papillo and Shapiro^15^ argue that increased heart rate demonstrates cognitive elaboration, that is, the amplification of basic information processing activities such as encoding, attention, and emotional processing through discussion, meta-cognition or imagination^16,17^. If narratives delivered in audiobook form require greater active mental simulation than watching videos, then we would expect to see increased heart rate for audio relative to video stories.

Electrodermal activity (EDA) is another physiological measure of engagement that is typically understood as an index of emotional arousal^18,19^. One of the key emotional centres in the brain, the amygdala, stimulates the adrenal medulla, releasing the hormone adrenaline and enhancing autonomic nervous system activity. One consequence is the constriction of sweat glands in the dermis which increase skin conductance. EDA, therefore, provides an indirect method for measuring emotional arousal^18^ that can be used to evaluate whether stories presented in either the auditory or visual modality differentially engage emotional responses.

Changes in body surface temperature have recently been suggested to correlate with mood and social context^20^. Since thermoregulation is biologically costly, IJzerman et al.^21^ argue that many social animals have evolved to share body warmth between themselves by directing blood towards the skin, and then huddling or engaging in skin-to-skin contact. Indeed, skin temperature on the hands increased by a fraction of a degree when participants watched film clips that produced positive, happy affect^22^ or engaged in positive social interactions^23^ and Kistler et al.^24^ found decreases in finger temperature in response to fear-inducing stimuli. By measuring body temperature at the wrist, we gain an independent physiological measure of potential differences between audiobook and video stories.

The question we asked here is whether a difference in the delivery channel would influence self report and physiological engagement with the narrative. Participants experienced the same set of eight scenes from fictional stories. For each story, participants either heard a passage from an audiobook, or saw a TV or movie adaptation of the same scene. For example, from the *Game of Thrones* book^25^ we chose the passage in which Arya witnesses her father’s beheading, and the same scene in the HBO adaptation^26^.

Stimuli can be informationally equivalent if the same information can be extracted from each and computationally equivalent if it can be extracted with the same effort^27,28^. Our stimuli are not equivalent in either of these senses. If a crowd scene is paused in the *Game of Thrones* scene, for example, a viewer could read off the colour of the clothing for every member in the shot. These are details that are not mentioned at all in the spoken narrative. But listeners to the audiobook are told, for example that Arya wondered why her sister looked happy. That information is either not present in the video at all, or has to be inferred from the actor’s emotions. Our claim therefore is not that the stimuli and informationally or computational equivalent, but that they are *narratively equivalent*, in the sense that the same story elements are present in each. The ways that stories are realised in video versus spoken word—the different information in each format and the different demands that they place upon the reader or viewer engaged in the narrative—are precisely what that we want to contrast experimentally.

While watching and listening to the stories, biometric sensors were used to measure physiological engagement via heart rate, electrodermal activity and body temperature. All of these physiological signals are also affected by physical activity, and so we used accelerometers on the sensors to track and account for body motion. After each story, participants answered twelve questions of a narrative engagement scale^29^ that quantified their immersion in the narrative, their attention to the story, their closeness to the characters and their sense of presence in the narrative world.

## Method

### Participants

109 participants were recruited from UCL’s subject pool and paid £10 for participation. Seven participants were excluded due to equipment failure or participant drop out. Of the 102 (41 M, 61F) who completed the experiment, their ages ranged from 18 to 55 with an average age of 29 years old (SD = 10.5). The sensors we used sometimes failed to record complete data, due to problems with the sensor, incorrect placement, the participant adjusting the wristband, and so on. The failure rate differed for different sensors as they were more or less susceptible to interruption and artifacts. After identifying and cleaning problematic recordings, we were left with 95 participants with complete heart rate and acceleration data, 76 with temperature data, and 62 with complete electrodermal activity data. This research was approved by the UCL Research Ethics Panel [#3828/003] in accordance with the Declaration of Helsinki. Participants provided written/electronic informed consent.

### Stimuli and materials

We chose eight works of fiction spanning four different genres. From classic literature we selected *Pride and Prejudice*^30^ and *Great Expectations*^31^; from action novels we chose *The Girl on the Train*^32^ and *The DaVinci Code*^33^; from crime fiction we chose *Hound of the Baskervilles* ^34^ and *The Silence of the Lambs*^35^ and from Science Fiction/Fantasy we chose *Alien: River of Pain*^36^ and *A Song of Ice and Fire* (Game of Thrones)^25^. Audiobooks were readings from the original texts, rather than acted out adapted audio plays, with sound effects and different actors. Videos were extracted from film or TV adaptations of the books. Each story was selected because they were well-known examples of their genres and because audio and video adaptations of the novel were available. For each, we selected an emotionally charged scene where the audio and video versions were as similar as possible. Though they covered the same events, due to their nature, the audiobooks tended to be slightly longer (M = 398 secs, SD = 210) than the video versions (M = 295 secs, SD = 117). A description of the eight scenes is included in the Supplemental Information.

The nature of narrative ‘engagement’ is notoriously difficult to define across contexts and disciplines^37^. For our purposes, we operationalised participants’ self-reported engagement in the stories by adapted the narrative engagement scale developed by Busselle and Bilandzic^29^, which is based on a mental models approach. This measure has the advantage of being validated across four dimensions of experiential engagement, which have been shown to be related to physiological measures^14^. The original scale referred to watching a program or film, and so we modified the language slightly to refer to stories that could be seen or heard. The scale is divided into four subscales with three questions relating to each: character engagement (e.g. “I understand why the main character thought and behaved as they did in the story”), narrative understanding (e.g. “I had hard time recognizing the thread of the story”), attentional focus (e.g. “I had a hard time keeping my mind on the story”), and narrative presence (e.g. “At times, the story was closer to me than the real world”).

### Procedure

Participants were informed about the experiment and gave their consent to take part. They were fitted with an Empatica E4 wrist sensor, which captured their heart rate (HR), electrodermal activity (EDA), wrist temperature and acceleration in 3 dimensions. They were led into a sound attenuated cubicle. The experiment was run on a PC, using the Gorilla online testing platform^38^ and participants wore headphones throughout. They first completed a short demographic questionnaire and a survey asking about their consumption of movies, books and audiobooks.

Participants were presented with eight stories, in a block of four audio books and four videos. Across participants we counterbalanced the order of these blocks, and which stories were presented as a video or audio book. In each trial, the participant first read a short synopsis of the plot and characters in the story so far, to give a context for the excerpt. They then watched the video onscreen or listened to the story while looking at a black screen. After presentation, participants reported whether or not they had experienced that excerpt before or not, and dragged a slider to indicate how familiar the characters were, and how familiar the scene was. Then they rated the 12 statements of the narrative engagement questionnaire, using a 7-point Likert scale that ranged from “strongly disagree” to “strongly agree.” The experiment took approximately an hour to administer. On completion the participants were debriefed, thanked and paid for their time.

### Data processing

Physiological data were aligned to stimulus and condition information and trimmed to trial durations using the Universal Time Coordinates that were recorded by the Empatica sensors and the Gorilla system. EDA measurements are typically susceptible to movement artifacts, and so we used the EDA Explorer algorithm^39^ to clean the EDA data using the acceleration data. The 3-dimensional acceleration vectors were then simplified into a single acceleration value that expressed movement magnitude in any direction.

All physiological measures were normalised for each participant. That is, the data were converted to a normal distribution by mean centring to zero and dividing by the standard deviation. This removed any baseline differences between individuals (e.g. their resting heart rates) and allowed us to focus on differences between story modalities within each participants’ data. Across the duration of the experiment, participants heart rates and body temperatures decreased overall, and their skin conductance changed (see SI Fig. 1), also they shifted in their ratings between blocks. These slower changes, presumable due to sitting still for 50 min, are factored out of our analyses (see SI) by modelling trial and block order. Additionally, we ran the analyses below on the first block only of the experiment, and the same pattern of condition differences were found, albeit with reduced evidence strength. The data are available from the Open Science Framework at https://osf.io/u452g/.

## Results

Participants reported that the videos were more engaging than the audiobooks by about 15% on average across our measures. Conversely, participants’ physiological measures showed higher levels for audiobooks rather than videos. In terms of raw measures, their average heart rate was higher when they were listening to audiobooks by about two beats a minute; they had a greater range of heart rate by about 4 beats per minute; their skin conductance (EDA) was higher by 0.02 micro Siemens and they were roughly a third of a degree warmer in their body temperature (0.34 °C).

**Figure 1:**
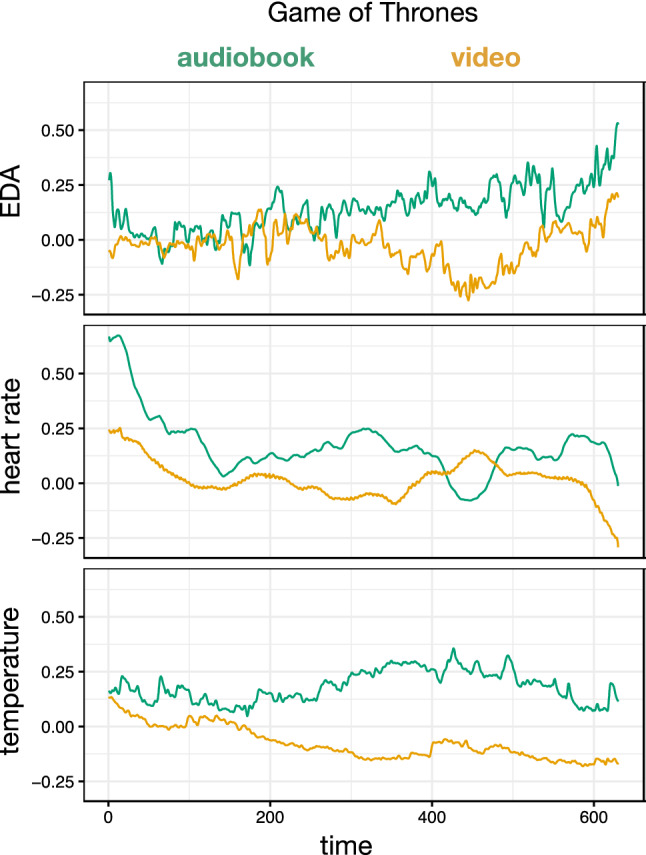
Time-course of physiological measures during the Game of Thrones story in two modalities.

Figure 1 shows an example of the time-course of our physiological measures for the *Game of Thrones* story in two modalities. Since the audiobook and video had different durations, we have plotted our measures as a function of the proportion of the story time. The differences shown for this item were echoed across all stories (see Supplementary Materials).

**Figure 2:**
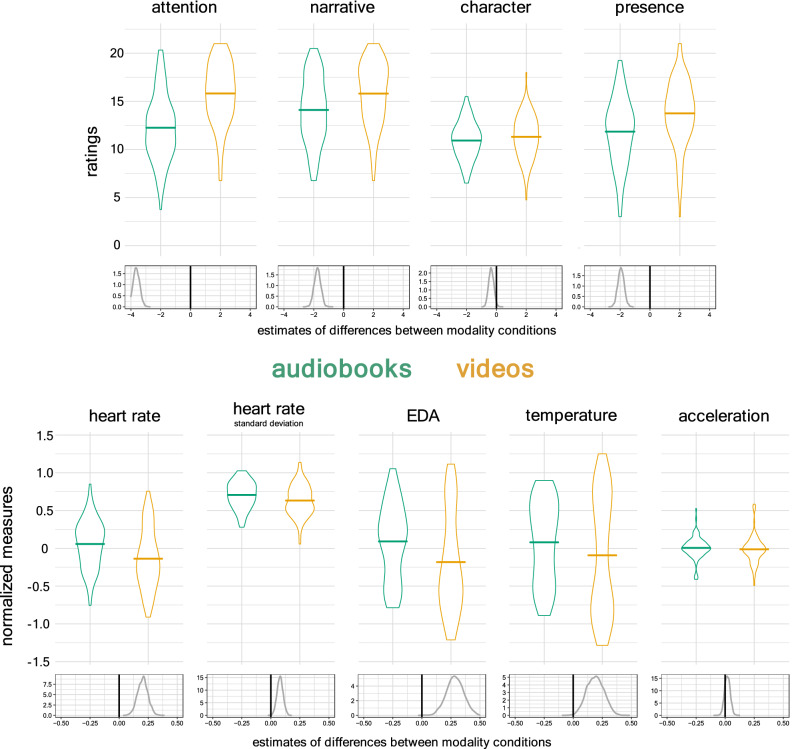
Participant self-report engagement ratings and physiological measures, split between story modalities. Beneath observed data are probability distributions for the estimated difference between modalities, derived from Bayesian mixed models. Grey shaded areas represent 95% credible intervals. When this region does not include the black line of zero difference, there is strong evidence supporting a difference between conditions.

Figure 2 presents the means and distributions for the participants’ self-report engagement ratings and normalized physiological measures, contrasting audio and video modalities. Beneath the observed data are probability distributions for the estimated differences between modalities. These estimates were derived from the posterior distributions given by Bayesian mixed models of our data^40^.

The Bayesian approach allowed us to directly quantify the effects of modality on behavioural and physiological measures and the strength of evidence in support of any differences, avoiding some of the problems associated with null hypothesis testing^41,42^. In the Supplementary Information, we also report more traditional ANOVAs analyses, which produced the same pattern of conclusions.

For each of the dependent variables reported below, we used mixed models with fixed effects for story modality, which was varied within participants, and block order and stimuli order, which were varied between participants as counterbalancing measures. We used random effects for participants, story, and trial number, to model changes in measures during the course of the experimental hour. We used R (version 3.4.3) the *rstanarm* package^43^ to model the data, and the psycho package^44^ to interpret it. In our models, we employed weakly informative priors that were scaled following the standard rstanarm procedure.

From 4,000 simulations, we generated estimates of the posterior distributions of the model parameter coefficients, which quantify the strength of the evidence that each experimental condition influenced behaviour. Full details of our models, priors, and all parameter estimates are given in the SI.

Here we report estimates of the differences between audio and video modalities. The full probability distributions of these difference estimates are shown in the bottom row of Fig. 2, below the observed data. Median estimates for the differences are given in the text below. We quantify the strength of the evidence in support of these difference using the Maximum Probability of Effect (MPE). This is the probability that the effect is positive or negative (depending on the median’s direction). In other words, the MPE directly quantifies the probability that the experimental condition had an effect. In Fig. 2 we also show 95% credible intervals for these estimates in grey (in other words, where the evidence suggests that there is 95% chance that the differences fall).

### Reported engagement

There was strong evidence that participants rated their engagement higher for videos rather than audiobooks. Summing the 3 questions for each subscale gave a value between 3 and 21. For their attention to the story, the median of the posterior distribution for the effect of the video condition was 3.62 points higher (MPE > 99.99%). For their engagement with the narrative, the median was 1.75 points higher (MPE > 99.99%). For their ratings of presence, the median was 1.95 points higher (MPE > 99.99%). For rated engagement with characters, there was a smaller but reliable difference of 0.35 points in favour of the videos (MPE = 98.23%). In other words, there was strong evidence that participants self-reported greater engagement for videos relative to audio stories for all measures of engagement.

### Physiological measures

The physiological evidence consistently demonstrated stronger responses for the audio relative to the video condition. The median estimate of normalized mean heart rates was 0.20 higher for audiobooks (MPE > 99.99%). The median standard deviation of heart rates was also greater by 0.08 (MPE = 99.75%). The median of the estimated EDA readings was 0.28 higher for audio books (MPE > 99.99%), and participants had a median estimate of normalised wrist temperature that was 0.19 higher for audiobooks (MPE = 99.35%).

One possible explanation for the higher physiological responses may be that participants were more physically active when listening to audiobooks—that is, they could have been fidgeting more, consistent with their self-reported lower engagement. To assess this, we examined the acceleration data from the Empatica sensors. There was no strong evidence that participants moved more during audiobooks, with the median estimated difference at 0.019 (MPE = 79.32%). The lack of an effect suggests that the differences in heart rate, EDA and temperature were not due to physical movement, but instead due to the emotional and cognitive differences in listening to audiobooks.

A second potential confound between the modalities was the fact that on average, audio scenes were longer than the equivalent video clips by approximately 100 s. To avoid cumulative differences in effort over time, we compare mean scores. Even so, if there were an upward linear trend with time, this could potentially inflate the difference with the longer clips showing larger effects. The data from all eight stories are shown in Supplemental Fig. 1. To investigate whether the longer clips affected the results, we trimmed all the physiological data to the length of the shortest modality (usually the video clip) and re-analysed the results. For each trial, the location of the trimming was chosen at random, and so the shortened data were equally likely to contain portions of the start or end of the full recording. The median estimate of normalized mean heart rates was 0.17 higher for audiobooks (MPE = 99.98%). The median standard deviation of heart rates was also greater by 0.06 (MPE = 97.52%). The median of the estimated EDA readings was 0.28 higher for audio books (MPE = 99.95%), and participants’ wrist temperature had a median estimated increase of 0.19 (MPE = 99.56%). In other words, the pattern of findings remained the same when the clips were truncated at a random location to the same duration across modalities.

### Familiarity

On a trial by trial basis, we computed how familiar the story and characters were to participants. Reported familiarity correlated positively and significantly with each of the four dimensions of reported engagement, but crucially, these correlations held equally for both audio and visual modalities (i.e. there was no statistical evidence that r values were significantly different between modalities by Zhou’s test). Reported familiarity did not correlate with any physiological responses, either for all items together, or for audio and video items separately. We concluded that while participants found familiar stories more engaging overall, this does not reveal anything about differences between audio and video narratives.

## Discussion

The term ‘engagement’ has different levels of meaning. In one sense, engagement relates to the richness of the experience while in another, is related to the degree of mental processing that generates that experience. In the case of audio versus video narratives, we have found a case where those two senses diverge.

Participants reported higher levels of engagement while watching video scenes compared to listening to audio scenes. They attended more, showed greater narrative understanding and reported greater narrative presence when watching video clips, suggesting that they not only found video narratives easier to comprehend, but also immersed themselves more fully in the world created by the video narratives. In other words, people found the videos more engaging according to their self-report. Interestingly, their implicit physiological measures told a different story. On average, heart rates were higher and more variable, ectodermal activity was greater and temperatures were raised when listening to audio narratives than when watching video narratives. These findings suggest that listening to audio stories engaged greater cognitive and emotional processing than watching videos.

If increased heart rate is truly an indicator of increased effort, these results are consistent with the hypothesis that listening to a story is a more active process, and therefore more cognitively and emotionally engaging than viewing the same story. In essence, the listener mentally simulates the narrative more so than viewers of the narrative, who more passively process the visualization provided by the video’s director.

Of course, this is a relative difference: understanding the narrative of a film certainly engages a range of cognitive and emotional processes. A recent study illustrated this by measuring brain activity while volunteers listened to stories that were either visually vivid, action-based, or emotionally charged^45^. All three story-types activated the temporal lobes and Broca’s area, as expected, but the interesting findings pertained to the differences between the stories. Specifically, visually vivid stories activated the occipito-parietal junction and the pre-cuneus, two regions associated with visuo-spatial processing. Action-based stories, in contrast, activated regions of premotor cortex while emotionally laden stories activated parts of the limbic system typically linked to affective responses, demonstrating that listening to stories engaged not only core “language regions” of the brain such as Broca’s area, but also recruited additional brain systems depending on the context. This is consistent with the notion that understanding a narrative involves mental simulation that retrieve the listener’s perceptual, motor, and affective knowledge through reactivation of the neural systems responsible for perception, action, and emotion.

Another fundamental difference between purely language-based stories and video is the presence of semantic ambiguity. Semantic ambiguity refers to the fact that most words in English have more than one meaning^46^ which means that listeners/readers are frequently resolving ambiguities, often without even noticing them. For example, in a sentence like “The woman made the toast with a new *microphone,*” the word “toast” is ambiguous—it could refer to cooked bread or a call to drink together. It is not until the word “microphone” is encountered that the meaning becomes clear. Although this appears to occur effortlessly, resolving ambiguity is a complex process involving multiple cognitive operations, supported by a set of brain regions including Broca’s area and posterior parts of the temporal lobe^47,48^. There is, however, less ambiguity in the video equivalent, where the image of a woman speaking into a microphone is clear from the outset. Even if they are unaware of it, the listener is working harder to understand the story than a person viewing a video would. As a result, listening to a story will be a more active and therefore more demanding process than watching the video.

There was also strong evidence from the physiological data for greater emotional engagement with the audio relative to the video versions of the stories. We observed significantly higher electrodermal activity (EDA) and skin temperatures when participants listened to audio narratives compared to when they watched the same narratives as video. These results demonstrate that when listening to audio stories, participants experienced greater arousal than when watching video stories.

Physiological measures such as heart rate, EDA and skin temperature are indirect indices of engagement. In each case the measure is a summary of autonomic nervous system activity which include additional factors beyond cognitive and emotional engagement (e.g. breathing, digestion, electrolyte concentrations, etc.—all of which relate to internal bodily states rather than external stimuli). Moreover, it is difficult to convincingly disentangle cognition from emotion for both practical and theoretical reasons. In practice, cognitive effort and emotional arousal have common effects on the autonomous nervous system that drive the physiological responses being measured here^12–14,18,19^. As a result, there is no way to disentangle the two based solely on heart rate or EDA measures. A more fundamental reason, however, is that emotions are simply not distinct from other forms of cognition—despite a widespread assumption to the contrary^49,50^, which is why we choose to interpret the current findings as evidence of “cognitive and emotional engagement.”

Of course, it is possible that these conclusions are specific to the particular narratives that we selected. Though they span genres, they are all cases of successful narratives, in the sense that people chose to record audio books and make film adaptions of them. What is remarkable, however, is that despite their differences in tone and content, the pattern of differences we found between conditions are replicated very consistently across items, and across the time course of items (see SI Fig. 3). An intriguing open question is the degree to which our findings are true of engagement in art forms that are non-linguistic (such as music versus music and dance), or stimuli that are linguistic but less narrative-driven, such as cooking instructions.

## Conclusion

We found that participants perceived themselves to be more concentrated and engaged while watching video narratives, but their physiological responses revealed more cognitive and emotional engagement while listening to audio narratives. Why do they feel more engaged if their bodies say otherwise? We suggest that spoken narratives require the participant to be an actively engaged listener, whereas videos deliver rich stimulation to a more passive viewer. The pictures in the listener’s mind may not be as vivid and as detailed as those onscreen, and so auditory narratives are rated explicitly as less engaging; yet the imaginative generation of those images requires greater cognitive and emotional processing, and so they are physiologically more engaging.

## Supplementary information

Supplementary information
