# Supplementary material for: Engagement in video and audio narratives: contrasting self-report and physiological measures
Source: Sci Rep. 2020 Jul 9;10:11298. doi: 10.1038/s41598-020-68253-2 (PMC7347852; doi:10.1038/s41598-020-68253-2)
Supplement: Supplementary file 1 — Supplementary information [file 41598_2020_68253_MOESM1_ESM.pdf]

Engagement in video and audio narratives: Contrasting self reported and physiological measures

Daniel C. Richardson, Nicole K. Griffin, Lara Zaki, Auburn Stephenson, Jiachen Yan, Thomas Curry, Richard Noble, John Hogan, Jeremy I. Skipper & Joseph T. Devlin

Supplementary Information

Excerpt synposes

Text in black was shown to participants prior to each story to contextualise the scene. Text in red is a brief summary of what the scene contained.

Da Vinci Code

While in Paris on business, Harvard symbologist Robert Langdon is informed that the elderly curator of the Louvre has been murdered inside the museum. Near the body, police have found a baffling cipher. Solving the enigmatic riddle, Langdon is stunned to discover it leads to a trail of clues hidden in the works of Da Vinci - clues visible for all to see, and yet ingeniously disguised by the painter. Langdon joins forces with a gifted French cryptologist, Sophie Neveu, and learns the late curator was involved in the Priory Of Sion - a secret society. In a breathless race through Paris, London and beyond, Langdon and Sophie match wits with a faceless power broker who appears to work for Opus Dei - a clandestine, Vatican-sanctioned Catholic organization believed to have long plotted to seize the Priory's secret. Unless Langdon and Sophie can decipher the labyrinthine puzzle in time, the Priory's secret - and a stunning historical truth - will be lost forever.

In this scene, Robert and Sophie view Leonardo’s Last Supper looking for the Holy Grail. After being told that the Challice is the symbol for a woman, Sophie notes that the painting is all men. She is astonished to discover that the person to Jesus’ right — in the place of honour — is indeed a woman: the Holy Grail. Sophie then learns that this is Mary Magdalen — Jesus’s wife.

Game of Thrones

Set in the fictional Seven Kingdoms of Westeros, Game Of Thrones follows several large and wealthy families as they struggle for power in an unstable political environment. The Starks, the Baratheons and the Lannisters form an uneasy alliance - peace in Westeros balances on a knife-edge. The Baratheons and the Starks are united through an old and lasting friendship between King Robert Baratheon and Warden of the North, Ned Stark. The Baratheons and the Lannisters are united through marriage - Robert is husband to Cercei Lannister, an influential and vengeful noblewoman. Over the course of many months the uneasy truce between these three houses begins to unravel. When Ned discovers a plot against the king, learning secrets about the legitimacy of Robert and Cersei's offspring in the process, his whole family are put at risk. Now that Ned's eldest daughter Sansa is engaged to Cersei's son Jeoffrey, and his youngest daughter Arya has escaped from the Lannisters to live in disguise as a peasant, Ned must reckon with the consequences of his actions.

In the scene, Arya climbs atop a statue to observe her father’s beheading above the crowd. She sees the high lords of the realm surrounding Lord Eddard, including Joffrey and his Queen Mother. Arya’s sister, Sansa, was also on the sept amongst the lords, looking happy. Spearman hold back the crowd. Ned Stark then confesses to his treason in front of a rowdy crowd and acknowledges Joffrey as the true king. The crowd threw stones while taunting Eddard. Joffrey then orders Eddard beheaded and the executioner drew a great sword and took Eddard’s head.

Great Expectations

Pip is an orphan living on the Kent marshes with his abusive sister and her husband, Joe Gargery, the village blacksmith. While exploring in the churchyard near the tombstones of his parents, Pip is accosted by an escaped convict. The convict scares Pip into stealing food for him, as well as a metal file to saw off the convict's chains. Returning with these the next morning, Pip discovers a second escaped convict, an enemy of the first one. Shortly afterward, both convicts are recaptured while fighting each other. Pip's pompous Uncle Pumblechook arranges for him to go to the house of a wealthy reclusive woman, Miss Havisham, to play with her adopted daughter, Estella. The house is strange and nightmarish.

In this scene, Pip arrives in a dressing room and meets Miss Havisham, a strange woman wearing an old bridal dress. She wants Pip to play for her entertainment. She has a sick fancy that she wants to see some play. When Pip doesn’t play immediately, she calls Estella — a young woman — into the room to play cards with Pip. Estella objects to playing with the labouring boy but is forced to play regardless. Estella insults Pip until Miss Havisham invites Pip to describe his impressions of Estella and he confides that he finds her both pretty and insulting.

SIlence of the Lambs

Clarice Starling is pulled from her training at the FBI Academy by Jack Crawford of the FBI's Behavioral Science Unit. He assigns her to interview Hannibal Lecter, a former psychiatrist and incarcerated cannibalistic serial killer, whose insight might prove useful in the pursuit of a serial killer nicknamed Buffalo Bill, who skins his female victims' corpses. When Buffalo Bill abducts a U.S. Senator's daughter named Catherine, Jack Crawford authorizes Clarice to offer Lecter a fake deal promising a prison transfer if he provides information that helps them find Buffalo Bill and rescue Catherine.

In this scene, Hannibal offers to trade Clarice for information. He will tell her about the case if she shares information about herself. Clarice admits her worst memory was the death of her father and describes the incident. She then reminds him of their quid pro quo agreement. Hannibal asks about the girl — was she large through the hips but flat chested? She was. Clarice also admitted that the woman had an insect inserted in her throat and Hannibal asks if it was a butterfly. Clarice is surprised because this information had not been shared. Hannibal then explains what Buffalo Bill wants: he is making a lady suit from his victims.

Alien

Whilst exploring deep space, many, many light-years away a crew of researchers, scientists and engineers discover an earth like planet which they decide to investigate before returning home. Having damaged their ship during the landing, a small crew breakaway from the main group and decide to explore the surrounding area for signs of habitation whilst their vessel is being repaired. During the course of their expedition, this small group of scientists come across a vast, derelict spaceship - an enormous horseshoe-shaped vessel - that looks like a promising find. As they travel deeper into the depths of this newly discovered vessel, they stumble across what remains of the ship's original crew.

In this scene, the explorers come across a room that includes eggs below a layer of mist. Soon after they realise there is movement within the eggs, the flaps atop an egg open. When the explorer takes a closer look, an alien leaps out of the egg and attacks.

Hound of the Baskervilles

Dr. James Mortimer asks Sherlock Holmes to investigate the death of his friend, Sir Charles Baskerville. Sir Charles was found dead on the grounds of his Devonshire estate, Baskerville Hall, and Mortimer now fears for Sir Charles' nephew and sole heir, Sir Henry Baskerville. Sir Charles' death was attributed to a heart attack, but Mortimer is suspicious, because the lord died with an expression of horror on his face.

In this scene, the client arrives to ask Sherlock and Holmes to investigate. He relates the tale of the Hound of the Baskerville.

Pride and Prejudice

This tale of love and values unfolds in the class-conscious England of the late 18th century. The five Bennet sisters - including strong-willed Elizabeth and young Lydia - have been raised by their mother with one purpose in life: finding a husband. When a wealthy bachelor takes up residence in a nearby mansion, the Bennets are abuzz. Amongst the man's sophisticated circle of friends, surely there will be no shortage of suitors for the Bennet sisters. But when Elizabeth meets up with the handsome and - it would seem - snobbish Mr. Darcy, a battle of the sexes begins. Despite a testy and confrontational start to their relationship Elizabeth learns that Mr. Darcy has taken steps to save her sister, Lydia, from personal disgrace at great personal expense. Over time Elizabeth's prejudices about Mr. Darcy are challenged and she gradually comes to recognise she may have misread the wealthy nobleman.

In this scene, Elizabeth and Mr. Darcy are on a walk, chaperoned by her sister Kitty with other members of the Bennet's walking behind. When Kitty leaves to call on a friend, Elizabeth takes her chance to thank Mr. Darcy for helping her sister. Darcy expresses surprise that the Aunt shared this information, but Elizabeth explains it was Lydia's thoughtlessness that revealed his help. Elizabeth reiterates her thanks from her family but Darcy says, if you must thank me, let it be from you alone. He then reveals he was thinking of Elizabeth and asks her for her feeling towards him. Elizabeth re-assures Darcy that she is pleased by his feelings, making them both rather happy.

Girl on a Train

Rachel Watson is an alcoholic reeling from the dissolution of her marriage to Tom, who left her for another woman, Anna. Rachel's drinking has caused her to lose her job; she frequently binges and has blackouts. While drunk, she often harasses Tom by phone and sometimes even in person, though she has little or no memory of these acts once she sobers up. Concealing her unemployment from her flatmate, Rachel sticks to her regular routine, taking the train to London every day as if she were still employed. Her train slowly passes her old house, occupied by an attractive couple - Scott and Megan. One day, Rachel witnesses Megan kissing a man who is not her husband. The next day, after a night of heavy drinking, Rachel awakens to find herself bloody and injured, with no memory of the night before but certain that she has done something she will regret. She later learns that Megan is missing and feared dead. Unable to trust her own memory, Rachel begins her own investigation, whilst simultaneously arousing the suspicions of the police.

In this scene, Rachel is interviewed by two police officers over her actions on the night Megan went missing. Rachel said she went to visit her ex-husband, Tom, but decided it wasn't a good idea and came home instead. The detectives interview Rachel, showing her a photo of Megan and asking if she knew this woman but Rachel denied seeing her. They ask if Rachel had been drinking and about her job, clearly establishing that she was lying — and Rachel knows this. But they then leave, asking her to contact them if she remembers anything important.

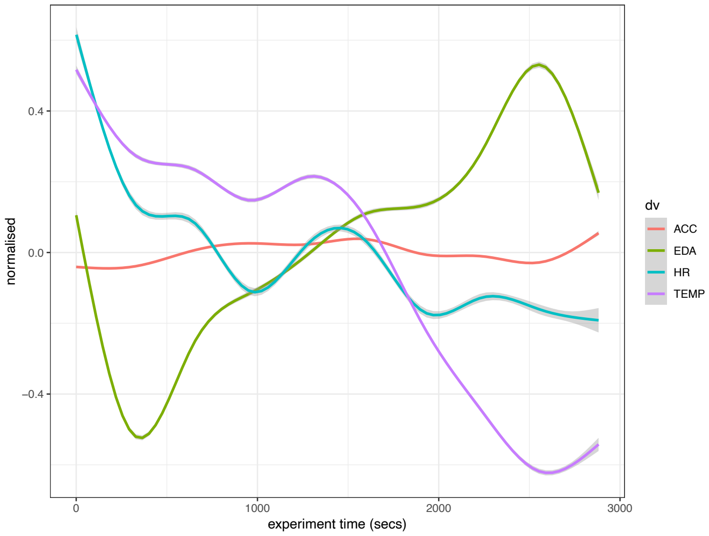

SI Figure 1. Physiological measures across the time course of the experiment.

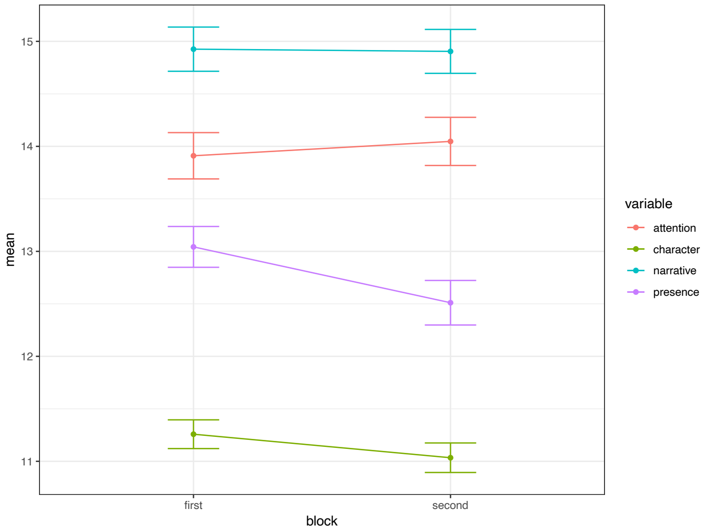

SI Figure 2. Ratings across the two blocks of the experiment.

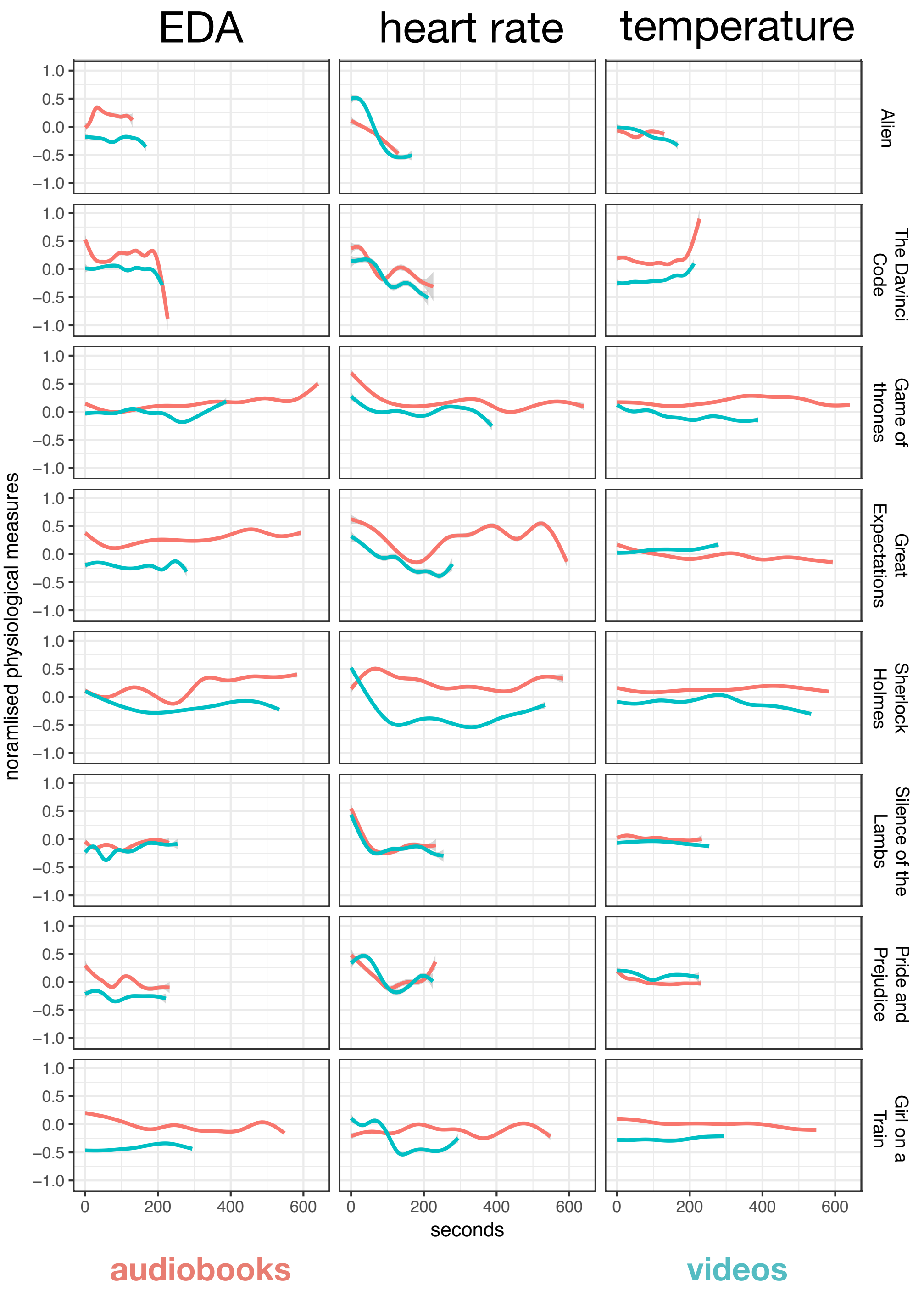

SI Figure 3. Timecourse of physiological measures for each story.

Analyses

We employed Bayesian Mixed Models for our analyses. In the main text, we report the probabilities that the model parameter for modality is non-zero, and the odds that modality influenced our measures. This information was dervived by fitting, for each dependent variable, a Markov Chain Monte Carlo model. Weakly informative priors from the Gaussian family were used that were scaled by the rstanarm package. We used random effects for the participant, the story and the trial number, and fixed effects for the modality, the modality order and the stimulus order, specified as:

Dependent variable ~ modality + modality order + stimulus order +
(1 | participant) + (1 | story) + (1 | trial number)

Analyses were run in R (v3.4.3) using the packages afex, rstanarm and psycho. In the tables below, we report information on the priors and detailed statistics for each models' parameters. We used the psycho package to calculate the MPEs and credible intervals for the marginal effects of modality conditions that are reported in the main text and plotted in figure 2.

We also report the results of 2 (modality) x2 (modality order) x2 (stimulus order) ANOVAs for readers who prefer frequentist analyses.

Engagement Ratings

Attention

Priors:

```
Intercept (after predictors centered)
 ~ normal(location = 0, scale = 10)
     **adjusted scale = 46.32

Coefficients
 ~ normal(location = [0,0,0], scale = [2.5,2.5,2.5])
     **adjusted scale = [11.58,11.58,11.58]

Auxiliary (sigma)
 ~ exponential(rate = 1)
     **adjusted scale = 4.63 (adjusted rate = 1/adjusted scale)

Covariance
 ~ decov(reg. = 1, conc. = 1, shape = 1, scale = 1)
```

Estimates of model parameters:

Variable	MPE	Median	MAD	Mean	SD	CI low	CI high
Intercept	100	12.47	0.63	12.47	0.64	11.15	13.67
modality	100	3.53	0.22	3.53	0.22	3.09	3.95
modality order	99.18	-1.29	0.55	-1.29	0.55	-2.32	-0.18
stimuli order	92.45	0.78	0.55	0.79	0.55	-0.26	1.89

Anova table:

Effect	df	MSE	F	p
stim_order	1,98	6.41	0.74	0.39
mod_order	1,98	6.41	2.33	0.13
stim_order:mod_order	1,98	6.41	0.08	0.78
modality	1,98	2.24	2.99	0.09
stim_order:modality	1,98	2.24	0.13	0.72
mod_order:modality	1,98	2.24	1.08	0.3
stim_order:mod_order:modality	1,98	2.24	6.89	0.01

Character

Priors:

```
Intercept (after predictors centered)
 ~ normal(location = 0, scale = 10)
     **adjusted scale = 28.56

Coefficients
 ~ normal(location = [0,0,0], scale = [2.5,2.5,2.5])
     **adjusted scale = [7.14,7.14,7.14]

Auxiliary (sigma)
 ~ exponential(rate = 1)
     **adjusted scale = 2.86 (adjusted rate = 1/adjusted scale)

Covariance
 ~ decov(reg. = 1, conc. = 1, shape = 1, scale = 1)
```

Estimates of model parameters:

Variable	MPE	Median	MAD	Mean	SD	CI low	CI high
Intercept	100	11.34	0.38	11.35	0.39	10.58	12.1
modality	98.15	0.34	0.15	0.34	0.16	0.01	0.66
modality order	92.13	-0.48	0.34	-0.48	0.35	-1.21	0.14
stimuli order	78.73	-0.26	0.33	-0.26	0.34	-0.89	0.44

Anova table:

Effect	df	MSE	F	p
stim_order	1,98	15.69	3.7	0.06
mod_order	1,98	15.69	6.33	0.01
stim_order:mod_order	1,98	15.69	0.09	0.76
modality	1,98	6.13	107.07	<.0001
stim_order:modality	1,98	6.13	0.02	0.9
mod_order:modality	1,98	6.13	0.07	0.79
stim_order:mod_order:modality	1,98	6.13	0	0.96

Narrative

Priors:

```
Intercept (after predictors centered)
 ~ normal(location = 0, scale = 10)
     **adjusted scale = 43.19

Coefficients
 ~ normal(location = [0,0,0], scale = [2.5,2.5,2.5])
     **adjusted scale = [10.80,10.80,10.80]

Auxiliary (sigma)
 ~ exponential(rate = 1)
     **adjusted scale = 4.32 (adjusted rate = 1/adjusted scale)

Covariance
 ~ decov(reg. = 1, conc. = 1, shape = 1, scale = 1)
```

Estimates of model parameters:

Variable	MPE	Median	MAD	Mean	SD	CI low	CI high
Intercept	100	14.03	0.58	14.03	0.59	12.82	15.13
modality	100	1.67	0.22	1.67	0.22	1.23	2.11
modality order	95.38	-0.93	0.56	-0.94	0.56	-1.99	0.16
stimuli order	96.8	1.06	0.57	1.05	0.56	-0.1	2.11

Anova table:

Effect	df	MSE	F	p
stim_order	1,98	15.5	6.42	0.01
mod_order	1,98	15.5	3.45	0.07
stim_order:mod_order	1,98	15.5	0.57	0.45
modality	1,98	5.14	28.94	<.0001
stim_order:modality	1,98	5.14	0.04	0.83
mod_order:modality	1,98	5.14	0.02	0.88
stim_order:mod_order:modality	1,98	5.14	0.12	0.73

Presence

Priors:

```
Intercept (after predictors centered)
 ~ normal(location = 0, scale = 10)
     **adjusted scale = 41.96

Coefficients
 ~ normal(location = [0,0,0], scale = [2.5,2.5,2.5])
     **adjusted scale = [10.49,10.49,10.49]

Auxiliary (sigma)
 ~ exponential(rate = 1)
     **adjusted scale = 4.20 (adjusted rate = 1/adjusted scale)

Covariance
 ~ decov(reg. = 1, conc. = 1, shape = 1, scale = 1)
```

Estimates of model parameters:

Variable	MPE	Median	MAD	Mean	SD	CI low	CI high
Intercept	100	12.32	0.59	12.32	0.58	11.14	13.42
modality	100	1.88	0.19	1.88	0.19	1.51	2.26
modality order	88.45	-0.73	0.6	-0.72	0.6	-1.89	0.44
stimuli order	66.05	-0.24	0.6	-0.24	0.6	-1.41	0.94

Anova table:

Effect	df	MSE	F	p
stim_order	1,98	18.84	0.03	0.87
mod_order	1,98	18.84	1.4	0.24
stim_order:mod_order	1,98	18.84	1.51	0.22
modality	1,98	4.89	39.17	<.0001
stim_order:modality	1,98	4.89	0.58	0.45
mod_order:modality	1,98	4.89	4.29	0.04
stim_order:mod_order:modality	1,98	4.89	1.12	0.29

Physiological Measures
Heart Rate

Priors:

```
Intercept (after predictors centered)
 ~ normal(location = 0, scale = 10)
     **adjusted scale = 6.73

Coefficients
 ~ normal(location = [0,0,0], scale = [2.5,2.5,2.5])
     **adjusted scale = [1.68,1.68,1.68]

Auxiliary (sigma)
 ~ exponential(rate = 1)
     **adjusted scale = 0.67 (adjusted rate = 1/adjusted scale)

Covariance
 ~ decov(reg. = 1, conc. = 1, shape = 1, scale = 1)
```

Estimates of model parameters:

Variable	MPE	Median	MAD	Mean	SD	CI low	CI high
Intercept	79.08	0.06	0.07	0.05	0.07	-0.09	0.2
modality	100	-0.17	0.05	-0.17	0.05	-0.27	-0.08
modality order	56	-0.01	0.05	-0.01	0.05	-0.1	0.09
stimuli order	75.05	0.03	0.05	0.03	0.05	-0.06	0.12

Anova table:

Effect	df	MSE	F	p
stim_order	1,91	0.02	0.02	0.9
mod_order	1,91	0.02	1.74	0.19
stim_order:mod_order	1,91	0.02	0.12	0.73
modality	1,91	0.2	9.16	0.003
stim_order:modality	1,91	0.2	1.85	0.18
mod_order:modality	1,91	0.2	13.87	0.0003
stim_order:mod_order:modality	1,91	0.2	0.87	0.35

Heart Rate Standard Deviation

Priors:

```
Intercept (after predictors centered)
 ~ normal(location = 0, scale = 10)
     **adjusted scale = 3.78

Coefficients
 ~ normal(location = [0,0,0], scale = [2.5,2.5,2.5])
     **adjusted scale = [0.95,0.95,0.95]

Auxiliary (sigma)
 ~ exponential(rate = 1)
     **adjusted scale = 0.38 (adjusted rate = 1/adjusted scale)

Covariance
 ~ decov(reg. = 1, conc. = 1, shape = 1, scale = 1)
```

Estimates of model parameters:

Variable	MPE	Median	MAD	Mean	SD	CI low	CI high
Intercept	100	0.66	0.04	0.66	0.04	0.58	0.75
modality	99.95	-0.08	0.03	-0.08	0.03	-0.13	-0.03
modality order	99.73	0.08	0.03	0.08	0.03	0.02	0.13
stimuli order	55.78	0	0.03	0	0.03	-0.06	0.05

Anova table:

Effect	df	MSE	F	p
stim_order	1,91	0.03	0.11	0.74
mod_order	1,91	0.03	5.75	0.02
stim_order:mod_order	1,91	0.03	0.21	0.65
modality	1,91	0.04	7.7	0.007
stim_order:modality	1,91	0.04	0.63	0.43
mod_order:modality	1,91	0.04	8.19	0.005
stim_order:mod_order:modality	1,91	0.04	4.78	0.03

EDA

Priors:

```
Intercept (after predictors centered)
 ~ normal(location = 0, scale = 10)
     **adjusted scale = 8.86

Coefficients
 ~ normal(location = [0,0,0], scale = [2.5,2.5,2.5])
     **adjusted scale = [2.21,2.21,2.21]

Auxiliary (sigma)
 ~ exponential(rate = 1)
     **adjusted scale = 0.89 (adjusted rate = 1/adjusted scale)

Covariance
 ~ decov(reg. = 1, conc. = 1, shape = 1, scale = 1)
```

Estimates of model parameters:

Variable	MPE	Median	MAD	Mean	SD	CI low	CI high
Intercept	80.03	0.12	0.15	0.13	0.15	-0.17	0.43
modality	100	-0.27	0.07	-0.27	0.07	-0.41	-0.12
modality order	68.55	-0.03	0.07	-0.03	0.07	-0.19	0.09
stimuli order	62.85	-0.02	0.08	-0.02	0.08	-0.17	0.13

Anova table:

Effect	df	MSE	F	p
stim_order	1,55	0.04	0.35	0.55
mod_order	1,55	0.04	0.89	0.35
stim_order:mod_order	1,55	0.04	0.06	0.81
modality	1,55	0.51	4.49	0.04
stim_order:modality	1,55	0.51	0.17	0.68
mod_order:modality	1,55	0.51	18.75	<.0001
stim_order:mod_order:modality	1,55	0.51	0.67	0.42

Temperature

Priors:

```
Intercept (after predictors centered)
 ~ normal(location = 0, scale = 10)
     **adjusted scale = 9.48

Coefficients
 ~ normal(location = [0,0,0], scale = [2.5,2.5,2.5])
     **adjusted scale = [2.37,2.37,2.37]

Auxiliary (sigma)
 ~ exponential(rate = 1)
     **adjusted scale = 0.95 (adjusted rate = 1/adjusted scale)

Covariance
 ~ decov(reg. = 1, conc. = 1, shape = 1, scale = 1)
```

Estimates of model parameters:

Variable	MPE	Median	MAD	Mean	SD	CI low	CI high
Intercept	58.3	0.03	0.16	0.03	0.18	-0.38	0.34
modality	99.2	-0.17	0.07	-0.17	0.07	-0.3	-0.03
modality order	88.28	0.09	0.07	0.09	0.07	-0.06	0.23
stimuli order	64.63	-0.03	0.07	-0.03	0.07	-0.17	0.11

Anova table:

Effect	df	MSE	F	p
stim_order	1,72	0.05	0.55	0.46
mod_order	1,72	0.05	6.99	0.01
stim_order:mod_order	1,72	0.05	0.51	0.48
modality	1,72	0.74	1.87	0.18
stim_order:modality	1,72	0.74	0.00	0.95
mod_order:modality	1,72	0.74	13.01	0.0006
stim_order:mod_order:modality	1,72	0.74	0.02	0.9

Acceleration

Priors:

```
Intercept (after predictors centered)
 ~ normal(location = 0, scale = 10)
     **adjusted scale = 3.20

Coefficients
 ~ normal(location = [0,0,0], scale = [2.5,2.5,2.5])
     **adjusted scale = [0.80,0.80,0.80]

Auxiliary (sigma)
 ~ exponential(rate = 1)
     **adjusted scale = 0.32 (adjusted rate = 1/adjusted scale)

Covariance
 ~ decov(reg. = 1, conc. = 1, shape = 1, scale = 1)
```

Estimates of model parameters:

Variable	MPE	Median	MAD	Mean	SD	CI low	CI high
Intercept	54.7	0	0.03	0	0.03	-0.05	0.05
modality	76.2	-0.02	0.02	-0.02	0.02	-0.06	0.03
modality order	55.68	0	0.02	0	0.02	-0.05	0.04
stimuli order	80.53	0.02	0.02	0.02	0.02	-0.02	0.06

Anova table:

Effect	df	MSE	F	p
stim_order	1,91	0.01	1.07	0.3
mod_order	1,91	0.01	0.03	0.87
stim_order:mod_order	1,91	0.01	2.19	0.14
modality	1,91	0.05	0.29	0.59
stim_order:modality	1,91	0.05	0.22	0.64
mod_order:modality	1,91	0.05	0	0.96
stim_order:mod_order:modality	1,91	0.05	0.04	0.85
